# The serotonergic nervous system of prolecithophorans shows a closer similarity to fecampiids than to triclads (Platyhelminthes)

**DOI:** 10.1002/jmor.21332

**Published:** 2021-02-19

**Authors:** Alexandra L. Grosbusch, Philip Bertemes, Bernhard Egger

**Affiliations:** ^1^ Research Unit, Evolutionary Developmental Biology Institute of Zoology, University of Innsbruck Innsbruck Austria

**Keywords:** 5‐HT, Adiaphanida, orthogon, Prolecithophora, Turbellaria

## Abstract

Prolecithophora is a poorly studied flatworm order belonging to the adiaphanidan clade, together with Tricladida and Fecampiida. The phylogenetic position of the three orders within this clade is not yet resolved. Additionally, no obvious synapomorphy other than an opaque epidermis could be found so far. In this study, the serotonergic nervous system of six different prolecithophoran species has been studied for the first time with a fluorescent immunocytochemical technique. We found that all six species show a similar pattern of the serotonergic nervous system. The typical prolecithophoran serotonergic nervous system consists of a cephalic ganglion in the anterior body part from which a pair of dorsal, ventral, and lateral longitudinal nerve cords originate. Furthermore, the three longitudinal nerve cords of one body side are connected to each other at the posterior body part by a conspicuous commissure. The ventral cords, which we consider the main cords, are most prominent and show double brain roots. A comparison of the nervous system within Adiaphanida shows clearly that prolecithophorans and fecampiids are much more similar in this regard than prolecithophorans and triclads.

## Graphical abstract

The nervous system in Prolecithophora has been studied for the first time using fluorescent immunocytochemistry. It consists of a brain and three longitudinal nerve cords, which are connected on either side of the body by the main lateral commissure. The ventral cords are considered as main cords and show two pairs of brain roots.

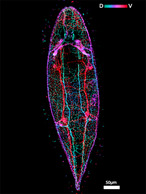

## 1 INTRODUCTION

Prolecithophora represents a group of small, free‐living, and mostly marine flatworms. Most species are quite opaque and have only one opening for male and female genitals (Jondelius et al., 2001; Karling, 1940; Norén & Jondelius, 2002; von Graff, 1913). Shape, location, and orientation of the pharynx are diverse and an important morphological feature for determination of the different families and species (Rieger & Sterrer, 1975; von Graff, 1904, 1913). Plagiostomidae von Graff, 1882 and Pseudostomidae von Graff, 1904 comprise the largest part of the taxon (Tyler et al., 2006–2019; WoRMS Editorial Board, 2020).

Pseudostomidae are oval‐shaped flatworms with a distinct brain capsule and an orogenital opening on the ventral side of the posterior body half. The pharynx is located in the posterior body part and points either to the posterior or to the anterior tip of the body. With only a few exceptions, pseudostomids have a ciliated groove and two pairs of eyes at the level of the brain (von Graff, 1913; Westblad, 1955).

Plagiostomidae mostly have a more oblong body shape and a light or no brain capsule. The oral and the genital opening are separated. The genital opening is located on the ventral side of the posterior body half, while the oral opening is on the ventral side near or at the front end of the body. The pharynx is located in the anterior body part and points towards the anterior tip of the body. A ciliated groove is rare among plagiostomids and most species have only one pair of eyes (von Graff, 1913; Westblad, 1956).

The nervous system of flatworms consists of a central nervous system (CNS), a peripheral nervous system (PNS), an autonomic pharyngeal‐stomatogastric nerve plexus, and a genital nerve plexus (Ehlers, 1985; Reuter & Gustafsson, 1995; Rieger et al., 1991). However, the pattern of the nervous system varies among the different flatworm groups. Substantial variation in number, location, and appearance of the longitudinal nerve cords can be observed. Thus, the terminology of main and minor cords has been suggested to avoid further confusion (Reuter et al., 1998; Reuter & Gustafsson, 1995). The main cords are the one pair of longitudinal cords that arises with prominent roots from the brain, they are formed of wide fiber bundles, and show the highest association with aminergic marker neurons. All other longitudinal nerve cords that are formed by thinner fiber bundles and show weaker contact with the brain are described as minor cords (Reuter et al., 1998; Reuter & Gustafsson, 1995). The introduction of main and minor cords affects the differentiation between CNS and PNS. The division into CNS and PNS can be predicated either on functional or morphological criteria. Based on function, the bilaterally symmetrical CNS encompasses the bilobed brain and the orthogon, one or several pairs of longitudinal nerve cords connected by transverse commissures, while the PNS consists of different kinds of plexuses (Reuter et al., 1998; Rieger et al., 1991). However, in the restricted morphological sense, the CNS contains besides the bilobed brain only the main cords, and the PNS consists of all the remaining minor cords and various nerve plexuses (Reuter et al., 1998). In the present study, the serotonergic nervous system was examined only morphologically, therefore the second definition of the CNS and PNS applies.

Not much is known about the organization of the nervous system in Prolecithophora. Kotikova and Timoshkin (1987) studied the nervous system of three different freshwater species, two belonging to the genus *Friedmaniella* Timoshkin & Zabrovskaja, 1985 and one to the genus *Porfirievia* Timoshkin, 1997 (both Baicalarctiinae Friedmann, 1933, Protomonotresidae Reisinger, 1924), on histological section series combined with a histochemical method for detection of cholinesterases. Furthermore, preliminary data on the distribution of catecholamines were obtained for *Pseudostomum quadrioculatum* (Leuckart, 1847) and *Cylindrostoma* sp. Joffe & Kotikova, 1991 (Joffe & Kotikova, 1991). In this study, we compare the pattern of the serotonergic nervous system of three pseudostomids (*Cylindrostoma fingalianum* (Claparède, 1861), *Cylindrostoma monotrochum* (von Graff, 1882), and *Monoophorum striatum* (Graff, 1878)) and three plagiostomids (*Plagiostomum koreni* Jensen, 187*8*, *Acmostomum dioicum* Metschnikoff, 1865, and *Vorticeros auriculatum* [Müller, 1784]) using an anti‐5‐hydroxytryptamine (5‐HT) fluorescent immunostaining method on whole‐mount adults.

## 2 MATERIAL AND METHODS

### 2.1 Animals

We collected adult specimens of all six prolecithophoran species [*Cylindrostoma fingalianum* (Claparède, 1861); *Cylindrostoma monotrochum* (von Graff, 1882); *Monoophorum striatum* (Graff, 1878); *Plagiostomum koreni* Jensen, 187*8*; *Acmostomum dioicum* Metschnikoff, 1865; *Vorticeros auriculatum* (Müller, 1784)] from brown algae in the port of Punat, Krk, Croatia (45°01′23”N 14°37′41″E) in March 2016, October 2016, 2017, May 2018, and in October 2019. The extraction and maintenance of the animals were made according to Grosbusch et al. (2019).

### 2.2 Immunocytochemistry

**FIGURE 1:**
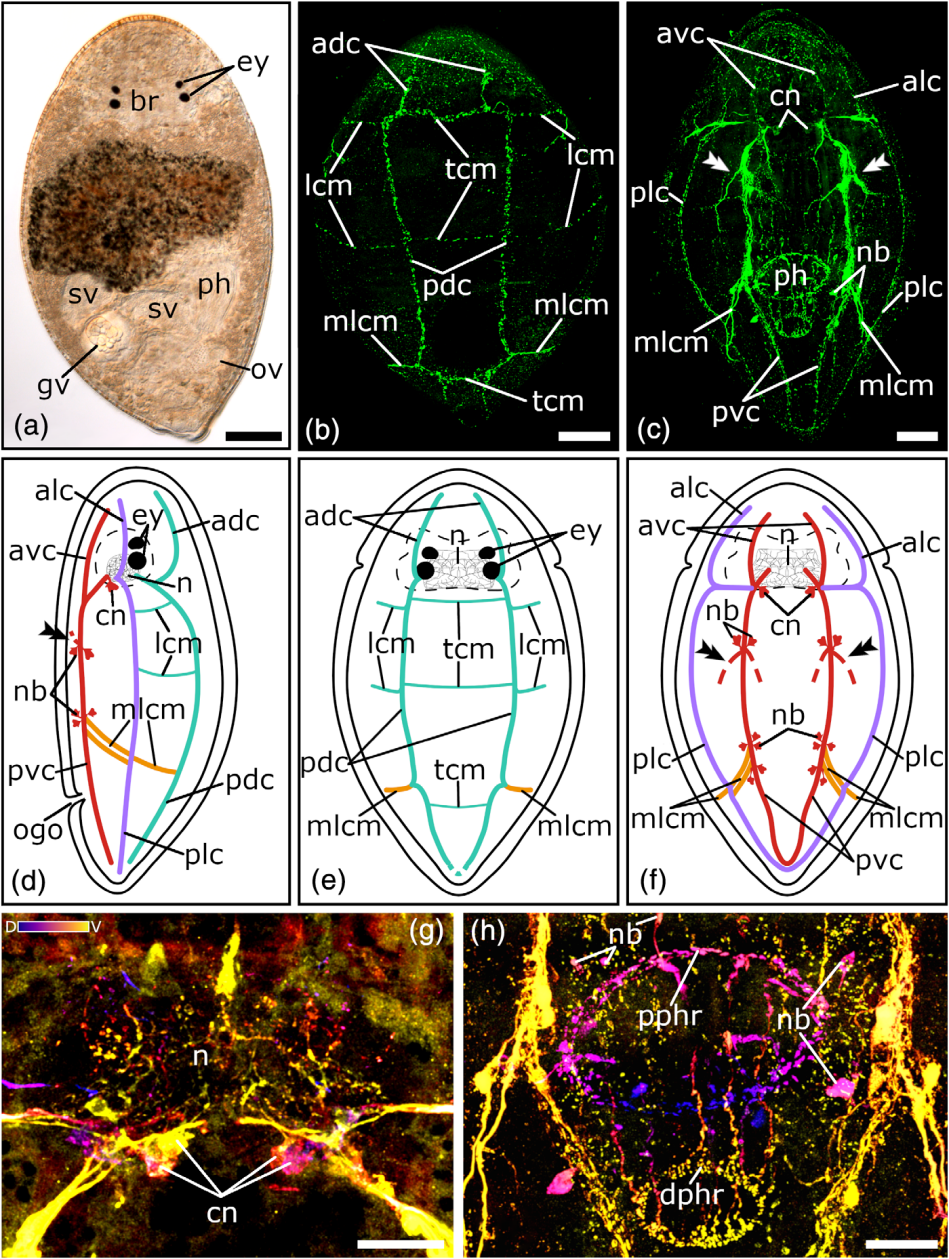
Serotonergic nervous system of *Cylindrostoma fingalianum*. (a) Dorsal view of a living adult animal in a whole mount squeeze preparation. (b–c) Confocal projection and (d‐f) schematic drawing of the nervous system in a (b, e) dorsal, (c, f) ventral, and (d) lateral view. (g, h) Depth‐color‐coded central projections of (g) the neuropil and (h) the pharyngeal nerve net, blue is more dorsal, yellow more ventral. The double arrowheads in (c, d, f) show the connection to the peripheral nerve plexus. Anterior is up for all animals, in (d) ventral is left. Scale bars: (a) 100 μm, (b, c) 50 μm, and (g, h) 25 μm. adc, anterior dorsal cords; alc, anterior lateral cords; avc, anterior ventral cords; br, brain; cn, cerebral neurons; dphr, distal pharyngeal nerve ring; ey, eyes; gv, granular vesicle; lcm, lateral commissures; mlcm, main lateral commissure; n, neuropil; nb, neuronal cell bodies; ogo, orogenital opening; ov, ovary; pdc, posterior dorsal cords; ph, pharynx; plc, posterior lateral cords; pphr, proximal pharyngeal nerve ring; pvc, posterior ventral cords; sv, seminal vesicle; tcm, transverse commissures

**FIGURE 2:**
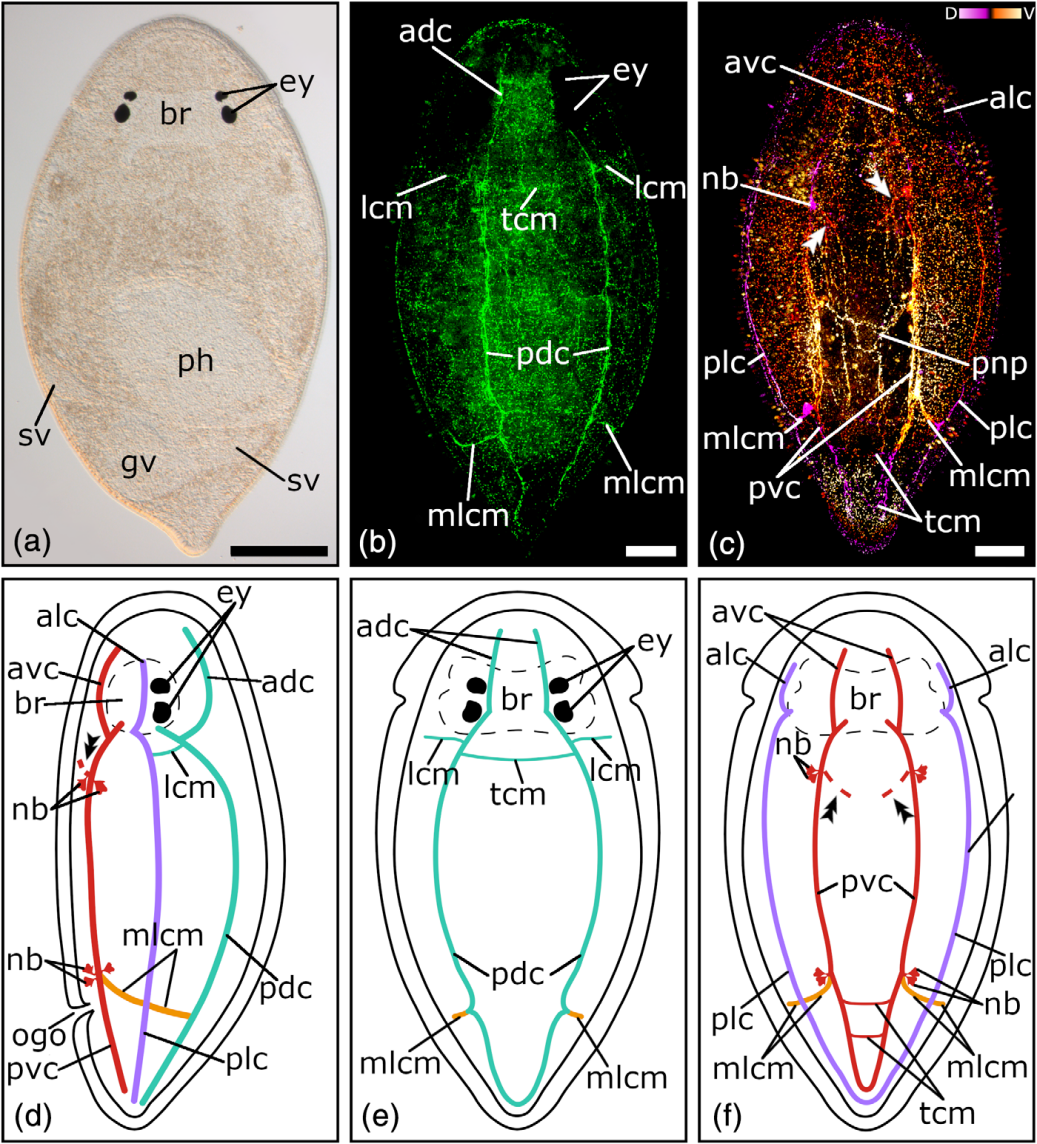
Serotonergic nervous system of *Cylindrostoma monotrochum*. (a) Dorsal view of a living adult animal in a whole mount squeeze preparation. (b) Confocal projection of a dorsal view of the nervous system. (c) Depth‐color‐coded projection of a ventral view of the nervous system, pink is more dorsal and yellow is more ventral. (d–f) Schematic drawing of the nervous system in a (e) dorsal, (f) ventral, and (d) lateral view. The double arrowheads in (c, d, f) show the connection to the peripheral nerve plexus. Anterior is up for all animals, in (d) ventral is left. Scale bars: (a) 200 μm and (b, c) 50 μm. adc, anterior dorsal cords; alc, anterior lateral cords; avc, anterior ventral cords; br, brain; ey, eyes; gv, granular vesicle; lcm, lateral commissures; mlcm, main lateral commissure; nb, neuronal cell bodies; ogo, orogenital opening; pdc, posterior dorsal cords; ph, pharynx; plc, posterior lateral cords; pnp, peripheral nerve plexus; pvc, posterior ventral cords; sv, seminal vesicle; tcm, transverse commissures

**FIGURE 3:**
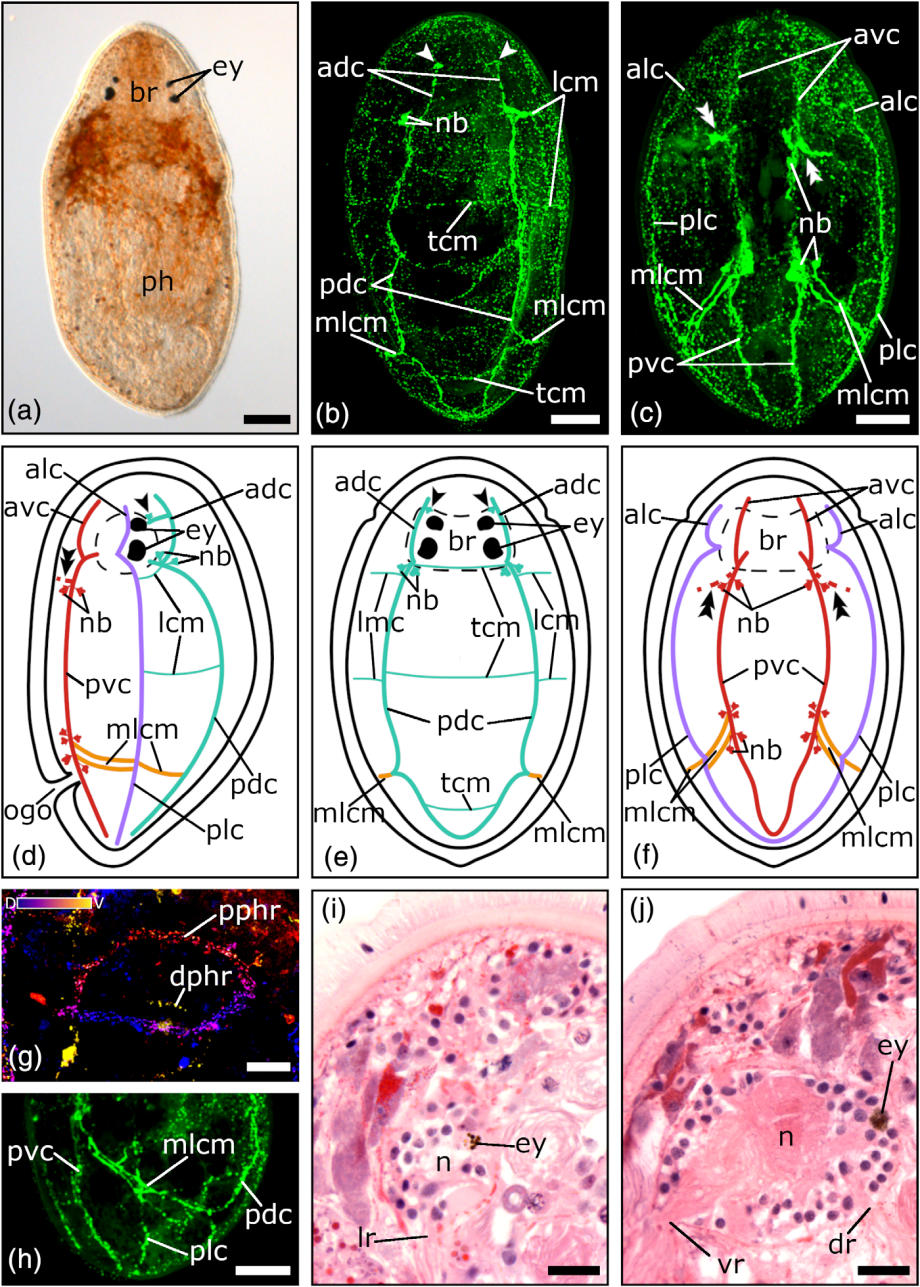
Serotonergic nervous system of *Monoophorum striatum*. (a) Dorsal view of a living adult animal in a whole mount squeeze preparation. (b–c) Confocal projection and (d–f) schematic drawing of the nervous system in a (b, e) dorsal, (c, f) ventral, and (d) lateral view. (g) Close‐up of a depth‐color‐coded central projection of the two pharyngeal nerve rings, blue is more dorsal, yellow more ventral. (h) Central projection of a lateral view of a specimen's posterior body half. (i–j) Sagittal histological sections showing brain roots of the lateral, dorsal, and ventral nerve cords. The single arrowheads in (b, d, e) show the pair of dorso‐medial nerve fibers, which runs from the dorsal nerve cords towards the foremost part of the brain. The double arrowheads in (c, d, f) show the connection to the peripheral nerve plexus. Anterior is up for all animals, in (d, h, i, j) ventral is left. Scale bars: (a) 100 μm, (b, c, h) 50 μm, and (g, i, j) 25 μm. adc, anterior dorsal cords; alc, anterior lateral cords; avc, anterior ventral cords, br, brain; dphr, distal pharyngeal nerve ring; dr, root of dorsal nerve cord; ey, eyes; lcm, lateral commissures; lr, root of lateral nerve cord; mlcm, main lateral commissure; n, neuropil; nb, neuronal cell bodies; ogo, orogenital opening; pdc, posterior dorsal cords; ph, pharynx; plc, posterior lateral cords; pphr, proximal pharyngeal nerve ring; pvc, posterior ventral cords; tcm, transverse commissures; vr, root of ventral nerve cord

**FIGURE 4:**
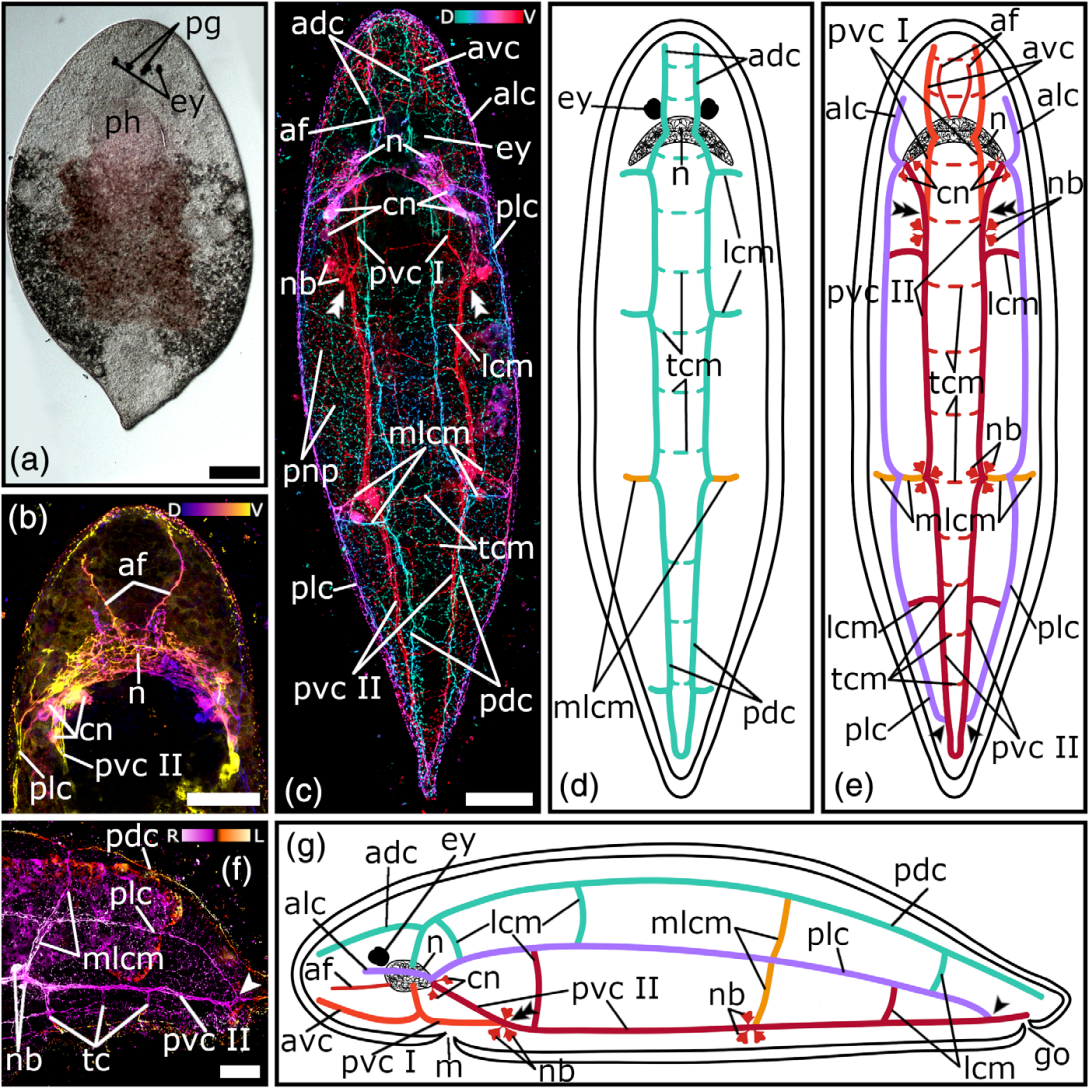
Serotonergic nervous system of *Plagiostomum koreni*. (a) Dorsal view of a living adult animal in a whole mount squeeze preparation. (b) Depth‐color‐coded central projection of the head region, blue is more dorsal, yellow more ventral. (c) Depth‐color‐coded projection of the whole animal, turquoise is more dorsal, red more ventral. (d, e, g) Schematic drawings of the nervous system in a (d) dorsal, (e) ventral, and (g) lateral view. (f) Depth‐color‐coded central projection of a lateral view of a specimen's posterior body half, pink is more right and yellow is more left. The dashed lines in (d) and (e) show transverse commissures, which on the one hand connect the nerve cords of a pair with each other and on the other hand form the connection to the peripheral nerve plexus. The single arrowheads in (e–g) mark the position at the posterior body part, where the lateral nerve cords connect to the ventral nerve cords. The double arrowheads in (c, e, g) show the connection site of the posterior part I and posterior part II of the ventral nerve cords. Anterior is up for animals in (a–e), in (f, g) anterior is left and dorsal is up. Scale bars: (a) 200 μm and (b, c, f) 50 μm. adc, anterior dorsal cords; af, anterior nerve fibers; alc, anterior lateral cords; avc, anterior ventral cords; cn, cerebral neurons; ey, eyes; go, genital opening; lcm, lateral commissures; m, mouth; mlcm, main lateral commissure; n, neuropil; nb, neuronal cell bodies; pdc, posterior dorsal cords; pg, pigments; ph, pharynx; plc, posterior lateral cords; pnp, peripheral nerve plexus; pvc I, posterior ventral cords part I; pvc II, posterior ventral cords part II; tcm, transverse commissures

**FIGURE 5:**
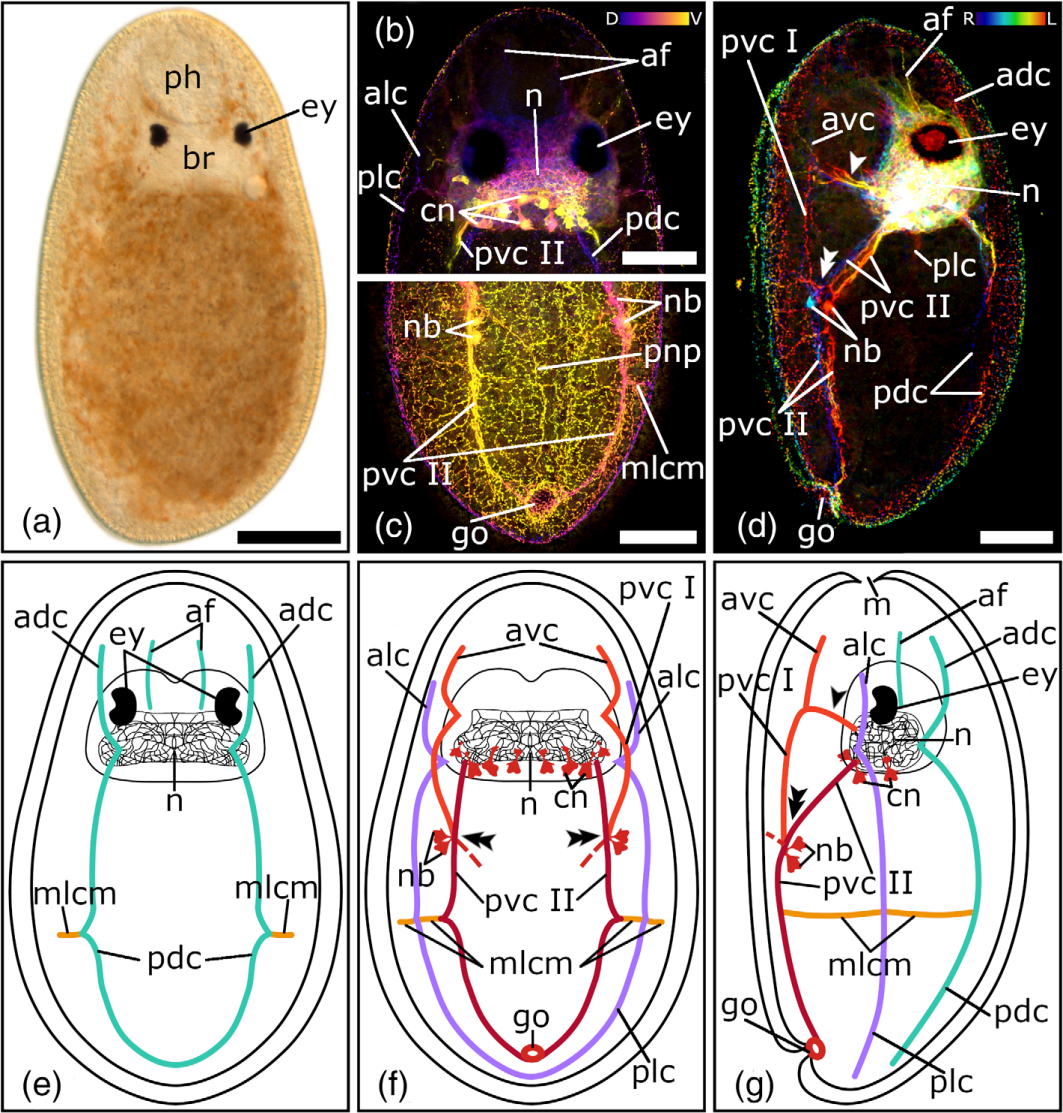
Serotonergic nervous system of *Acmostomum dioicum*. (a) Dorsal view of a living adult animal in a whole mount squeeze preparation. (b–d) Depth‐color‐coded (b) central projection of the head, (c) ventral projection of the posterior body, (d) lateral projection of the whole animal. In (b) and (c) blue is more dorsal, yellow more ventral, in (d) blue is more right, red more left. (e–g) Schematic drawings of the nervous system in a (e) dorsal, (f) ventral, and (g) lateral view. The dashed lines in (f, g) indicate the connection to the peripheral nerve plexus. The single arrowheads in (d, g) mark the brain root of the anterior part and the posterior part I of the ventral nerve cords. The double arrowheads in (d, f, g) show the connection site of the posterior part I and posterior part II of the ventral nerve cords. Anterior is up for all animals, in (d, g) ventral is left. Scale bars: (a) 100 μm and (b–d) 50 μm. adc, anterior dorsal cords; af, anterior fibers; alc, anterior lateral cords; avc, anterior ventral cords; br, brain; cn, cerebral neurons; ey, eyes; go, genital opening; m, mouth; mlcm, main lateral commissure; n, neuropil; nb, neuronal cell bodies; pdc, posterior dorsal cords; ph, pharynx; plc, posterior lateral cords; pnp, peripheral nerve plexus; pvc I, posterior ventral cords part I; pvc II, posterior ventral cords part II

**FIGURE 6:**
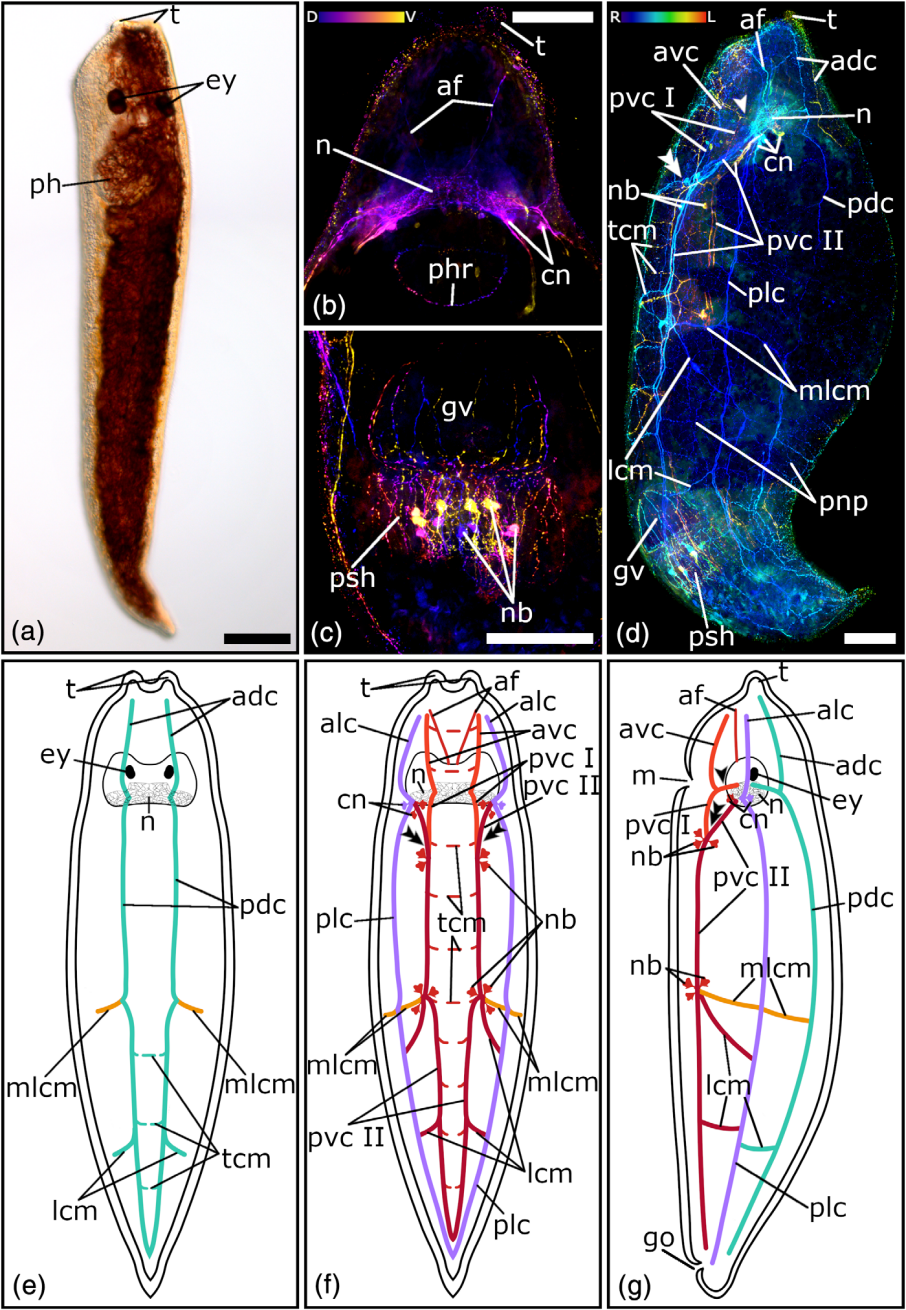
Serotonergic nervous system of *Vorticeros auriculatum*. (a) Dorsal view of a living adult animal in a whole mount squeeze preparation. (b–d) Depth‐color‐coded central projection of (b) the head, and (c) the tail, (d) lateral projection of the whole animal. In (b) and (c) blue is more dorsal, yellow more ventral, in (d) blue is more right, red more left. (e–g) Schematic drawings of the nervous system in a (e) dorsal, (f) ventral, and (g) lateral view. The single arrowheads in (d, g) mark the brain root of the anterior part and the posterior part I of the ventral nerve cords. The double arrowheads in (d, f, g) show the connection site of the posterior part I and the posterior part II of the ventral nerve cords. The dashed lines in (e, f) show transverse commissures, which on the one hand connect the nerve cords of a pair with each other and on the other hand form the connection to the peripheral nerve plexus. Anterior is up for all animals, (d, g) ventral is left. Scale bars: (a–d) 100 μm. adc, anterior dorsal cords; af, anterior fibers; alc, anterior lateral cords; avc, anterior ventral cords; cn, cerebral neurons; ey, eyes; go, genital opening; gv, granular vesicle; lcm, lateral commissures; m, mouth; mlcm, main lateral commissure; n, neuropil; nb, neuronal cell bodies; pdc, posterior dorsal cords; ph, pharynx; phr, pharyngeal nerve ring; plc, posterior lateral cords; pnp, peripheral nerve plexus; psh, penis sheath; pvc I, posterior ventral cords part I; pvc II, posterior ventral cords part II; t, tentacles; tcm, transverse commissures

For staining, nine adult *C. fingalianum* (Figure 1(a)), five adult *C. monotrochum* (Figure 2(a)), 12 adult *M. striatum* (Figure 3(a)), six adult *P. koreni* (Figure 4(a)), 14 adult *A. dioicum* (Figure 5(a)), and seven adult *V. auriculatum* (Figure 6(a)) were used. *Cylindrostoma monotrochum* and *M. striatum* were stained according to Grosbusch et al. (2019). Four individuals of *V. auriculatum* were stored at −20°C before staining; two in ethanol and two in aceton. Furthermore, for *A. dioicum*, *C. fingalianum*, *P. koreni*, and *V. auriculatum* some modifications of the staining protocol were made. After incubation with the first antibody (rabbit anti‐5HT, Sigma‐Aldrich), animals were washed for 24 h with PBS‐T_x_ (1× phosphate buffered saline with 0.1% Triton X‐100, Sigma‐Aldrich), and then incubated for 1 h at room temperature (RT) in the secondary fluorescein isothiocyanate‐conjugated (FITC) goat anti‐rabbit antibody (DAKO, Denmark), diluted 1:250 in BSA‐T_x_ (PBS‐T_x_ with 1% bovine serum albumin, Carl Roth, Germany). Specimens were washed again with PBS‐T_x_ for five days at RT and five nights at 4°C in darkness.

### 2.3 Histological sections and staining

Specimens were embedded in Technovit resin and semi‐thin sectioned at 3 μm thickness using a microtome (Reichert‐Jung Autocut 2040) and stained using a standard hematoxylin & eosin (H&E) protocol (see Grosbusch et al., 2019 for details).

### 2.4 Microscopy and visualization

Live and slightly squeezed animals as well as histological sections were observed and documented using a Leitz Diaplan or a Leica DM 5000 B light microscope equipped with a Motic Moticam 1080 camera or a Leica DFC 490 camera, respectively. Confocal stacks of fluorescently stained animals were made with a Leica TCS SP5 II confocal microscope. Images were analyzed and processed with the open‐source software Fiji v. 1.52j (Schindelin et al., 2012). The look‐up tables “Ice”, “ICA 3”, “physics” and “mpl plasma”, included in Fiji, were used to create depth‐color‐coded images. Schemes were drawn with the open‐source software Inkscape v. 0.92 (https://inkscape.org) and picture editing was done using the open‐source software GIMP v. 2.10.8 (https://gimp.org).

## 3 RESULTS

### 3.1 The serotonergic nervous system in Pseudostomidae

#### 3.1.1 The brain

A weak immunoreactivity in the neuropil of the brain is visible in one individual of *C. fingalianum* (Figure 1(g)). In the area between the two eye pairs, thin nerve fibers form an interlacing texture (Figure 1(d–g)). On each side of the posterior part of the brain, four to six large perikarya of cerebral neurons are grouped together near the roots of the ventral and lateral nerve cords. No immunoreactivity in the region of the brain is visible in any stained specimens of *M. striatum* or *C. monotrochum*.

#### 3.1.2 The orthogon: longitudinal nerve cords

All three pseudostomid species show three prominent longitudinal nerve cord pairs: a pair of ventral nerve cords, a pair of lateral nerve cords, and a pair of dorsal nerve cords (Figures 1(b–f); 2(b–f); 3(b–f). Each nerve cord arises with its own root from the posterior part of the brain, near the posterior eye pair and splits up in an anterior and a posterior part, which runs either towards the anterior or posterior part of the body (Figures 1(b–g); 2(b–f); 3(b–f, i–j)). In *M. striatum*, from the anterior part of the dorsal nerve cords, a pair of dorso‐medial nerve fibers runs towards the foremost part of the brain (Figure 3(b, d, e)). Furthermore, in *M. striatum*, dorsal cords are the thinnest longitudinal nerve cords (3.8 μm ± 0.9 μm, *n* = 6), while lateral cords (5 μm ± 1.1 μm, *n* = 4) are only slightly thicker than ventral cords (4.8 μm ± 1.4 μm; *n* = 5). In *C. monotrochum* and *C. fingalianum*, however, ventral cords are the thickest (*C. monotrochum*: 4.8 μm ± 1.2 μm, *n* = 4; *C. fingalianum*: 4.7 μm ± 1.2 μm, *n* = 6) and dorsal cords (*C. monotrochum*: 3.4 μm ± 1.4 μm, *n* = 4; *C. fingalianum*: 2.7 μm ± 0.5 μm, *n* = 6) and lateral cords (*C. monotrochum*: 3.4 μm ± 1.0 μm, *n* = 4; *C. fingalianum*: 2.5 μm ± 0.6 μm, *n* = 6) are almost equally thick.

In *C. monotrochum* and *M. striatum*, the dorsal nerve cords form a loop in the posterior tip of the body (Figures 2(e); 3(b, e)), while in *C. fingalianum*, no caudal loop of the dorsal nerve cords can clearly be seen (Figure 1(b, e)). Ventral and lateral nerve cords form a caudal loop in all three species (Figures 1(c, f); 2(c, f); 3(c, f)). Neither dorsal, nor ventral, nor lateral nerve cords show a frontal loop (Figure 1(b, c, e, f); 2(b, c, e, f); 3(b, c, e, f)).

#### 3.1.3 The orthogon: commissures

Due to several transverse commissures between the longitudinal nerve cords, the serotonergic nervous system shows the typical orthogonal pattern. However, one prominent commissure, which connects the posterior parts of the dorsal, the lateral, and the ventral nerve cords of one side of the body, can be observed in all three species (Figures 1(b–f); 2(b–f); 3(b–f, h)). Because it connects the three different longitudinal nerve cords, we name it the main lateral commissure (mlcm). In *C. monotrochum*, it consists continuously of a single nerve fiber (Figure 2(b–f)), whereas in *C. fingalianum* and *M. striatum*, the connecting fibers are paired between the ventral and the lateral cords (Figures 1(c, d, f); 3(c, d, f, h)). Some weaker commissures are visible on the dorsal side of the animals (Figures 1(b, e); 2(b, e); 3(b, e)). In *M. striatum* and *C. fingalianum*, three thin transverse commissures (tcm), one right behind the brain, one in the middle of the body, and one in the caudal part of the body, connect the two cords of the dorsal pair. On either side of the body two lateral commissures (lcm), one right behind the brain and one in the middle of the body, run from the dorsal nerve cords towards the lateral nerve cords (Figures 1(b, d, e); 3(b, d, e)). In *C. monotrochum*, only one transverse commissure between the two dorsal nerve cords is visible right behind the brain and also only one lateral commissure can be seen on either side of the body right behind the brain running from the dorsal nerve cords towards the lateral nerve cords (Figure 2(b, d, e)). On the ventral side, only *C. monotrochum* shows two transverse commissures at the caudal end connecting the two ventral nerve cords (Figure 2(c, f)). Clusters of highly immunoreactive perikarya at connection sites of the ventral nerve cords are striking in all three species (Figures 1(c, d, f); 2(c, d, f); 3(c, d, f)). *Monoophorum striatum* is the only species that shows clusters of immunoreactive perikarya at the posterior part of the brain on the dorsal nerve cords (Figure 3(b, d, f)).

#### 3.1.4 The peripheral nerve plexus

A coarse meshwork of thin nerve fibers forms a peripheral nerve plexus in all three species. This peripheral nerve plexus is best stained on the ventral side in *C. monotrochum*, in which a regular pattern can be observed (Figure 2(c)). In the other two species, only few and scattered fibers of the peripheral nerve plexus can be seen (data not shown). All three longitudinal nerve cord types show a connection to the peripheral nerve plexus but those starting from the ventral nerve cords on the middle of the anterior body half are the most conspicuous (Figures 1(c, d, f); 2(c, d, f); 3(c, d, f)).

#### 3.1.5 Pharyngeal and genital nerve plexus

The pharyngeal nerve plexus is best stained in *C. fingalianum* (Figure 1(c, h)). Consisting of a larger proximal nerve ring (diameter: 68.7 μm ± 16.1 μm, *n* = 6) connected by 10–12 thin longitudinal nerve fibers to a smaller distal nerve ring (diameter: 27.6 μm ± 9.8 μm, *n* = 4), it has the shape of a basketball hoop (Figure 1(c, h)). The perikarya of the connecting nerve fibers are located anterior to the proximal ring (Figure 1(h)). In *M. striatum*, the staining of the pharyngeal nerves is weak. Mainly the larger proximal ring (diameter: 103 μm ± 25.3 μm, *n* = 4) is visible, but in some individuals a weak distal ring (diameter: 58.1 μm ± 9.1 μm, *n* = 3) can also be seen (Figure 3(g)). In *C. monotrochum*, no staining in the area of the pharynx could be detected. None of the three species shows a staining of the genital nerve plexus.

### 3.2 The serotonergic nervous system in Plagiostomidae

#### 3.2.1 The brain

The neuropil of the brain is stained in all three plagiostomid species. The strongest staining can be seen in *P. koreni* (Figure 4(b)). Behind the eyes, interlaced nerve fibers are arranged in a crescent moon shape (Figure 4(b–e)). At each tip of the sickle, a cluster of five to six large perikarya of cerebral neurons surround the roots of the lateral and ventral nerve cords (Figure 4(b, c, e, g)). While in *P. koreni* only the neuropil can be seen, in *A. dioicum* and *V. auriculatum* a cloud‐like texture fills the whole area of the brain (Figures 4(b, c); 5(b, d); 6(b, d)). In *A. dioicum* the interlaced nerve fibers of the neuropil are arranged in a trapezoidal shape (Figure 5(b, e, f)) and the perikarya of cerebral neurons are not restricted to the area of the roots of lateral and ventral nerve cords but they cover also the area in between (Figure 5(b)). In total, 14–16 perikarya can be seen at the posterior part of the neuropil (Figure 5(b, f)). In *V. auriculatum*, the nerve fibers of the neuropil build a looser meshwork than in the other two plagiostomids (Figure 6(b, d)) and form a trapezoidal shape, which is flatter and more elongated than in *A. dioicum* (Figure 5(b)). A cluster of four to five large perikarya of cerebral neurons around the roots of the lateral and ventral nerve cords can also be seen in *V. auriculatum* (Figure 5(b)).

#### 3.2.2 The orthogon: longitudinal nerve cords

The three plagiostomid species show the same three pairs of longitudinal nerve cords as the pseudostomids (Figures 4(c–e, g); 5(d–g); 6(d–g)). In all three species ventral cords are the thickest longitudinal nerve cords (*P. koreni*: 4.6 μm ± 1.2 μm, *n* = 5; *A. dioicum*: 5.9 μm ± 1.4 μm, *n* = 10; *V. auriculatum*: 6.6 μm ± 2.5 μm, *n* = 3). While in *P. koreni* and *A. dioicum* the lateral cords are the thinnest (*P. koreni*: 2.7 μm ± 0.8 μm, *n* = 5; *A. dioicum*: 2.4 μm ± 0.3 μm, *n* = 5), in *V. auriculatum*, this applies to the dorsal cords (3.7 μm ± 1.5 μm, *n* = 3).

The dorsal nerve cords originate (as so‐called roots) from the middle of the brain (Figures 4(c, d, g); 5(d, e, g); 6(d, e, g)), while the lateral nerve cords arise on both sides of the posterior part of the brain (Figures 4(c, e, g); 5(d, f, g); 6(d, f, g)). Both, lateral and dorsal nerve cords, split into an anterior and a posterior part, which extend towards the anterior or posterior part of the body, respectively (Figures 4(c–g); 5(d, f, g); 6(d–g)). In all three species, the dorsal nerve cords form a loop in the caudal end of the body, but no frontal loop can be seen in the anterior body part (Figures 4(c, d); 5(e); 6(e)). The lateral nerve cords show the same characteristics as the dorsal nerve cords with the exception that in *P. koreni*, the posterior parts of the lateral nerve cords do not form a loop at the caudal body part, but they connect to either one of the ventral nerve cords (Figures 4(c, e, f, g); 5(f, g); 6(f, g)).

The ventral nerve cords show the same peculiar appearance in all three species (Figures 4(c, e, g); 5(c, d, f, g); 6(d, f, g)). While dorsal and lateral nerve cords show one pair of roots in the brain and are divided into an anterior and a posterior part, the ventral nerve cords have two brain root pairs (one pair per side) and can be subdivided into three parts, an anterior part, a posterior part I, and a posterior part II (Figures 4(c, e, g); 5(d, f, g); 6(d, f, g)). The first roots of the ventral nerve cords originate at the middle of the brain and split into two parts, the anterior part and the posterior part I (Figures 4(c, e, g); 5(d, f, g); 6(d, f, g)). While the anterior part extends towards the frontal tip of the body, the posterior part I runs towards the posterior body part and connects to the posterior part II of the ventral nerve cords at about the middle of the anterior body half (Figures 4(c, e, g); 5(d, f, g); 6(d, f, g)). The posterior part II has its roots right next to those of the lateral nerve cords on both sides of the posterior part of the brain (Figures 4(c, e, g); 5(b, d, f, g); 6(d, f, g)). Then, they run towards the caudal tip of the body and form a loop (Figures 4(c, e); 5(c, f); 6(f)).

At the front of the brain, all three species have a pair of thin nerve fibers named “anterior nerve fibers”, which run towards the frontal tip of the animal. While in *P. koreni* and *V. auriculatum* they resemble a V‐shape (Figures 4(b, c, e); 6(b, f)), in *A. dioicum* they run parallel to each other (Figure 5(b, e)).

#### 3.2.3 The orthogon: commissures

Same as in pseudostomids, the three longitudinal nerve cords of one body side are connected by the main lateral commissure (mlcm; Figures 4(c–g); 5(c, e–g); 6(d–g)). This commissure is prominent in *P. koreni* and *V. auriculatum*, but is only weakly stained in a few individuals of *A. dioicum*. In addition, it is the only commissure that can be seen in *A. dioicum*. However, *P. koreni* and *V. auriculatum* show some additional lateral commissures (lcm) on the dorsal and the ventral side (Figures 4(c–e, g), 6(d–g)). *P. koreni* has three additional lateral commissures on the dorsal side (Figure 4(c, d, g)). One is right behind the brain, the second is near to the middle of the body and the third is at the posterior body part near the tip of the tail. On the ventral side, *P. koreni* has two additional lateral commissures; one is in the anterior body part, near the connection site of posterior part I and posterior part II of the ventral nerve cords and the other one is in the middle of the posterior body part (Figure 4(c, e)). *V. auriculatum* has one additional lateral commissure on the dorsal side at the middle of the posterior body part (Figure 6(d, e, g)). On the ventral side, *V. auriculatum* has two additional lateral commissures (Figure 6(d, f, g)). One originates at the same site on the ventral nerve cord as the main lateral commissure, but then runs more diagonally and stops at the lateral nerve cord. The second one is located at the middle of the posterior body half, same as in *P. koreni*.

*P. koreni* and *V. auriculatum* also show transverse commissures on the dorsal and the ventral side, which connect the two cords of the dorsal and ventral pair of nerve cords, respectively (Figures 4(c–f); 6(d–f)). *P. koreni* shows numerous transverse commissures on the dorsal (11–13) and the ventral (12–14) side (Figure 4(c–f)). *V. auriculatum* only has few (2–4) transverse commissures on the dorsal side which are restricted to the posterior body part, while on the ventral side numerous (9–10) transverse commissures connect the two ventral nerve cords (Figure 6(d–f)). As with pseudostomids, clusters of highly immunoreactive perikarya at the connection sites of the ventral nerve cords can be seen in all three plagiostomids.

#### 3.2.4 The peripheral nerve plexus

A dense, varicose meshwork forms a peripheral nerve plexus over the whole body in *A. dioicum*, *P. koreni*, and *V. auriculatum* (Figures 4(c); 5(c); 6(d)). In *P. koreni* and *V. auriculatum*, the peripheral nerve plexus is at the same level as the cords of the orthogon (Figures 4(c); 6(d)). Thus, there is a clear connection between the peripheral nerve plexus and the orthogon.

#### 3.2.5 Pharyngeal and genital nerve plexus

Two individuals of *V. auriculatum* show a weak staining in the area of the pharynx. Behind the brain, immunoreactive nerve cells, all about the same size, form a pharyngeal nerve ring (Figure 6(b)). The other two plagiostomids do not show any staining of the pharyngeal innervation.

The male genital organ in *V. auriculatum* is surrounded by a nerve plexus, which has the shape of a jellyfish and consists mainly of longitudinal nerve fibers (Figure 6(c, d)). While the longitudinal nerve fibers around the granular vesicle are far apart, those surrounding the penis sheath are close together. There are large perikarya that are arranged in a ring around the penis sheath. However, the male genital organ was stained only in two individuals. Neither in *P. koreni*, nor in *A. dioicum* a staining was seen in the region of the genital organs. However, in *A. dioicum* the genital opening at the posterior body part on the ventral side is surrounded by a ring built of numerous nerve cells (Figure 5(c, d, f, g)). This nerve ring shows a connection to the ventral nerve cords.

## 4 DISCUSSION

### 4.1 The brain

Neuronal cell bodies surrounding a fibrillar neuropil form the basic structure of the cephalic ganglion in flatworms (Reuter & Gustafsson, 1995; Reuter & Halton, 2001; Richter et al., 2010; Rieger et al., 1991). In four out of six studied prolecithophoran species, we could observe a staining in the brain. In general, the stained brain texture consists of a central, fibrillar neuropil and four to eight pairs of neuronal cell bodies, which are located at the posterior border of the brain, near the roots of the ventral and lateral nerve cords. The brain structure is consistent with the structure described previously in prolecithophorans, except for the distribution of the neuronal cell bodies, which referred to the entire periphery of the brain in the preceding studies (Joffe & Kotikova, 1991; Kotikova & Timoshkin, 1987). However, this could be explained by the different staining methods that were used. A study of the nervous system of a monogenean parasite has revealed that the distribution of nerve cell bodies in the brain shows differences depending on whether cholinergic, aminergic, or peptidergic substances were used (Maule et al., 1990). Furthermore, some studies on the nervous system of two triclad species (*Polycelis tenuis* Ijima, 1884 and *Dendrocoelum lacteum* [Müller, 1774]) as well as on a polyclad species (*Theama mediterranea* Curini‐Galletti et al., 2008), a macrostomorphan species (*Microstomum lineare* [Müller, 1773]) and a proseriate species (*Bothriomolus balticus* Meixner, 1938), showed that 5‐HT immunoreactive (5‐HT‐IR) cell bodies were limited to the posterior border of the brain while RF‐amide immunoreactive cell bodies were distributed over the entire periphery of the brain (Bertemes et al., 2020; Joffe & Reuter, 1993; Reuter et al., 1986, 1996).

A closer look at the stainings shows that the shape of the 5‐HT‐IR neuropil differs between species, with the shape of the neuropil being most extraordinary in *P. koreni*. In the latter, the neuropil has the shape of a sickle while *C. fingalianum, A. dioicum*, and *V. auriculatum* show a more or less trapezoid shape. However, the neuropil of *C. fingalianum* and *A. dioicum* is broader than that of *V. auriculatum*. In triclads and polyclads, a correlation of the shape and position of the cephalic ganglion and the body shape, especially the shape of the head, has been noticed (Lang, 1884; Quiroga et al., 2015; Sluys, 1989). *C. fingalianum* and *A. dioicum* have a droplet‐shaped body while *P. koreni* and *V. auriculatum* have more oblong bodies. Furthermore, the head shape of *P. koreni* is slightly narrower than that of the other three species. Thus, it is possible that also here the body shape correlates with the different shapes of the neuropil.

The fact that a staining of the 5‐HT‐IR neuropil could only be seen in one pseudostomid species and in all three plagiostomid species is most probably due to a capsule surrounding the brain in some species. This layer of extracellular matrix was first discovered in representatives of the Polycladida, but by now it is also known for some species of proseriates, prorhynchids, rhabdocoels, and prolecithophorans (Böhmig, 1891; Lang, 1884; Rieger et al., 1991). Böhmig (1891) was the first to describe a brain capsule in prolecithophorans. In the pseudostomids, a distinct brain capsule was observed, which consists of either two or three layers. In the plagiostomids, the brain is also surrounded by a membrane, but by consisting of a loose network of fine fibers, it shows a different structure to that in pseudostomids (Böhmig, 1891; Karling, 1940; Ritter‐Záhony, 1908). This is a likely reason why the 5‐HT‐IR neuropil was stained in all three plagiostomids, but only in one pseudostomid in this study.

The typical flatworm brain consists of two bilaterally symmetrical lobes that are mainly connected by one or several neuropilar commissures (Ehlers, 1985; Rieger et al., 1991). These commissures are most pronounced in triclads, giving the brain the shape of a butterfly (Cebrià, 2007; Mäntylä et al., 1998; Reuter et al., 1996, 1998; Vila‐Farré et al., 2010). However, in macrostomorphans, polyclads, proseriates, rhabdocoels, as well as in prolecithophorans a transverse commissure is completely missing, and the two halves of the brain are fused together. The brain has an incision at the anterior and posterior part that indicates the division (Joffe & Reuter, 1993; Kotikova et al., 2002; Kotikova & Timoshkin, 1987; Ladurner et al., 2005; Quiroga et al., 2015; von Graff, 1882). Our results agree with these observations, as they do not show a clear separation of the brain halves either. In fecampiids, a division of the brain is never mentioned (Joffe & Kotikova, 1991; Raikova et al., 2017). A study of the cholinergic nervous system of the fecampiid *Notentera ivanovi* Joffe et al., 1997 showed the shape of the rather rudimentary brain, which is reminiscent of a six‐pointed star (Raikova et al., 2017). The illustration of the brain of another fecampiid species, *Urastoma cyprinae* (von Graff, 1882), is similar to the prolecithophoran brain. It is of a trapezoid shape and the roots of the longitudinal nerve cords show a similar distribution to that seen in our stainings (Joffe & Kotikova, 1991).

### 4.2 The orthogon: definition of main cords

Nerve cords must show certain characteristics to be called main cords: they must be thick fiber bundles emerging with prominent roots in the brain and they must be associated with more neurons or groups of neurons than other nerve cords (Joffe & Reuter, 1993; Reuter et al., 1998). In the studied species, the ventral nerve cords are the thickest with the exception of *M. striatum*, where the lateral nerve cords are slightly thicker than the ventral nerve cords. Additionally, the ventral nerve cords show the highest association with 5‐HT‐positive neuronal cell bodies in all studied species, including *M. striatum*. The ventral nerve cords also show the strongest brain roots in those species, where the neuropil has been successfully stained. All former studies of the prolecithophoran nervous system describe the ventral longitudinal nerve cord pair as the strongest and with prominent roots in the brain (Böhmig, 1891; Karling, 1940; Ritter‐Záhony, 1908). Thus, in almost all studied species, the ventral nerve cords fulfill the criteria for main nerve cords. Since the structure of the nervous system as well as the location of the main nerve cords are similar within most flatworm families and even orders (Joffe & Reuter, 1993; Reuter et al., 1998; Reuter & Gustafsson, 1995), we suggest that the ventral cords take over the role of the main cords in all prolecithophoran species, even in *M. striatum*.

### 4.3 The orthogon: longitudinal nerve cords

We found that all six prolecithophoran species show a pair of dorsal, ventral, and lateral nerve cords with their roots in the posterior brain part. In the three plagiostomid species, two brain roots of the ventral cords could be shown. With only a few exceptions, all prior descriptions of the nervous system of prolecithophoran species describe the following posterior longitudinal nerve cords originating from four roots in the posterior half of the brain: dorsal, ventral, lateral, and ventrolateral. An early study was based on histological sections of and near the brain and did not examine the eventual merging of the ventral and ventrolateral cords into the ventral cord (Böhmig, 1891). However, a study on the morphology of eight different prolecithophoran species (three Pseudostomidae, three Plagiostomidae, one Protomonotresidae, one Scleraulophoridae Marcus, 1950) showed that in one pseudostomid species the ventrolateral (in our terminology: posterior ventral cords) and the ventral (in our terminology: anterior ventral cords) nerve cords unite at a thickening of the ventrolateral nerve cord and thus can be described as one single cord. Although this condition was not clearly visible in the remaining species, the author assumes that it is the same for all pseudostomids (Karling, 1940). In three protomonotresid species, it has been shown that the ventral cords as well as the dorsal cords have two roots in the brain (Kotikova & Timoshkin, 1987). For *C. monotrochum*, two pairs of posterior dorsal nerve cords have been described where the roots of one pair arise right behind the eyes (Ritter‐Záhony, 1908). Although no connection of the two cords has been described, it is possible that the two dorsal nerve cords merge into one single dorsal cord. In the present study, a possible second root of the dorsal cords was detected only in *M. striatum*. Therefore, it can be hypothesized that the presence of a double brain root of the ventral nerve cords occurs in all prolecithophoran species, while the presence of two brain roots of the dorsal nerve cords cannot be certainly confirmed. Data on more individuals and more species as well as other staining methods of the nervous system is required for this purpose.

In Protomonotresidae, ventral nerve cords fan out at the anterior body part and merge with the peripheral nerve plexus, while lateral and dorsal nerve cords connect to each other on either side of the body (Kotikova & Timoshkin, 1987). In the present study, the fate of the anterior parts of the longitudinal nerve cords could not clearly be observed as it is difficult to differentiate between peripheral nerve plexus and lateral nerve cords at the periphery of the animals. However, this indicates that longitudinal nerve cords may thin out at the anterior body end and connect with the peripheral nerve plexus. Posterior parts of dorsal and ventral nerve cords have been observed to form a caudal loop in all studied species except for *C. fingalianum*, where no caudal loop of the dorsal nerve cords could be clearly seen. In three protomonotresid species, ventral and dorsal nerve cords also build a caudal loop (Kotikova & Timoshkin, 1987). We hypothesize that dorsal and ventral nerve cords always build a caudal loop in prolecithophorans, even in *C. fingalianum*. Lateral nerve cords have been observed to form a posterior loop in all studied species except for *P. koreni*, where they connect to the corresponding ventral nerve cords. Kotikova and Timoshkin (1987), however, write that the posterior parts of the lateral nerve cords are splitting up and connect to the corresponding dorsal and ventral nerve cords in protomonotresids, so apparently the pattern of the posterior part of the lateral nerve cords varies in prolecithophorans.

### 4.4 The orthogon: commissures

A peculiar characteristic of the orthogon was found in all species investigated here. On both sides of the posterior body part, a prominent continuous commissure, the main lateral commissure, connects the dorsal, ventral, and lateral nerve cord. A similar connection has been found in Protomonotresidae, as it has been reported that the lateral nerve cords divide into two branches and connect to the corresponding ventral and dorsal nerve cords (Kotikova & Timoshkin, 1987). However, the location of the connection varies between the families. In the investigated plagiostomids, the main lateral commissure is located near the middle of the body and in the investigated pseudostomids, it is located in the last fifth of the body. For protomonotresids, it is described that the connection lies at the posterior end of the body (Kotikova & Timoshkin, 1987). Interestingly, in *C. monotrochum* the main lateral commissure consists of one single 5‐HT‐IR nerve fiber, whereas in its congener *C. fingalianum* and also in *M. striatum* its ventral part is made of two 5‐HT‐IR nerve fibers.

### 4.5 The orthogon: comparison to other Adiaphanida

In the two closely related orders Fecampiida and Tricladida, the ventral cords are also the main cords (Reuter et al., 1998; Reuter & Halton, 2001). With a few exceptions, the orthogon in triclads consists of two pairs of longitudinal nerve cords (marginal and ventral) and commissures are equally spaced all over the body (Cebrià, 2007; Mäntylä et al., 1998; Reuter et al., 1996; Vila‐Farré et al., 2010). In fecampiids, however, the orthogon includes three longitudinal cord pairs (dorsal, ventral, and lateral) and on either side of the posterior body part, the corresponding dorsal, ventral, and lateral nerve cords are connected to each other (Joffe & Kotikova, 1991; Raikova et al., 2017). In *U. cyprinae*, even double brain roots of the ventral and dorsal nerve cords were found (Joffe & Kotikova, 1991). Thus, we can conclude that the pattern of the prolecithophoran orthogon is much more similar to that of fecampiids than to that of triclads. However, this could be related to the fact that prolecithophorans are much more similar to fecampiids in body size and body thickness than to most triclads.

### 4.6 Pharyngeal and genital nerve plexus

Mainly, the pharyngeal nerve plexus in Platyhelminthes consists of one or two nerve rings, a distal and a proximal ring, which are connected by several longitudinal nerve fibers (Rieger et al., 1991). While we could find this exact pattern in *C. fingalianum*, in *M. striatum* and *V. auriculatum* only a weak staining of pharyngeal nerve rings could be seen. Observations from previous studies agree with our results. However, also a connection between the ventral longitudinal nerve cords and the pharyngeal nerve plexus was described (Böhmig, 1891; Karling, 1940; Kotikova & Timoshkin, 1987). The latter could not be detected in our stainings. Nonetheless, a connection between the main cords and the pharyngeal nerve plexus is often mentioned in flatworms (Rieger et al., 1991). In triclads, the pharyngeal nerve plexus shows a unique pattern. A prominent outer cylindrical nerve plexus and a delicate inner cylindrical nerve plexus innervate the pharyngeal wall (Cebrià, 2007; Reuter et al., 1996, 1998; Rieger et al., 1991; Vila‐Farré et al., 2010). Among fecampiids, which usually lack a pharynx, *U. cyprinae* is an exception and has a rudimentary pharynx (Joffe et al., 1997; Rohde et al., 1994; von Graff, 1913). The pharynx of *U. cyprinae* is innervated by six to seven neurons which build two nerve rings and connecting longitudinal nerve fibers (Joffe & Kotikova, 1991).

It is unclear why the pharyngeal and genital nerve plexus is only stained in a few individuals of some species in our stainings. One explanation is that flatworms often produce a dense mucus layer around their body when they are stressed, which might result in reduced penetration of the antibody into the animal (Rieger et al., 1991). Another reason could be the choice of the targeted part of the nervous system: in some representatives of Tricladida, a difference in stainings with peptidergic and aminergic substances can be noticed. For example in *D. lacteum* the immunoreactivity to RF‐amide in the genital organs was more prominent than that to 5‐HT, while in *P. tenuis* and *Planaria torva* (Müller, 1773) the genital organs could only be stained by using peptidergic substances (Mäntylä et al., 1998; Reuter et al., 1996).

## 5 CONCLUSION AND SYSTEMATIC CONSIDERATIONS

**FIGURE 7:**
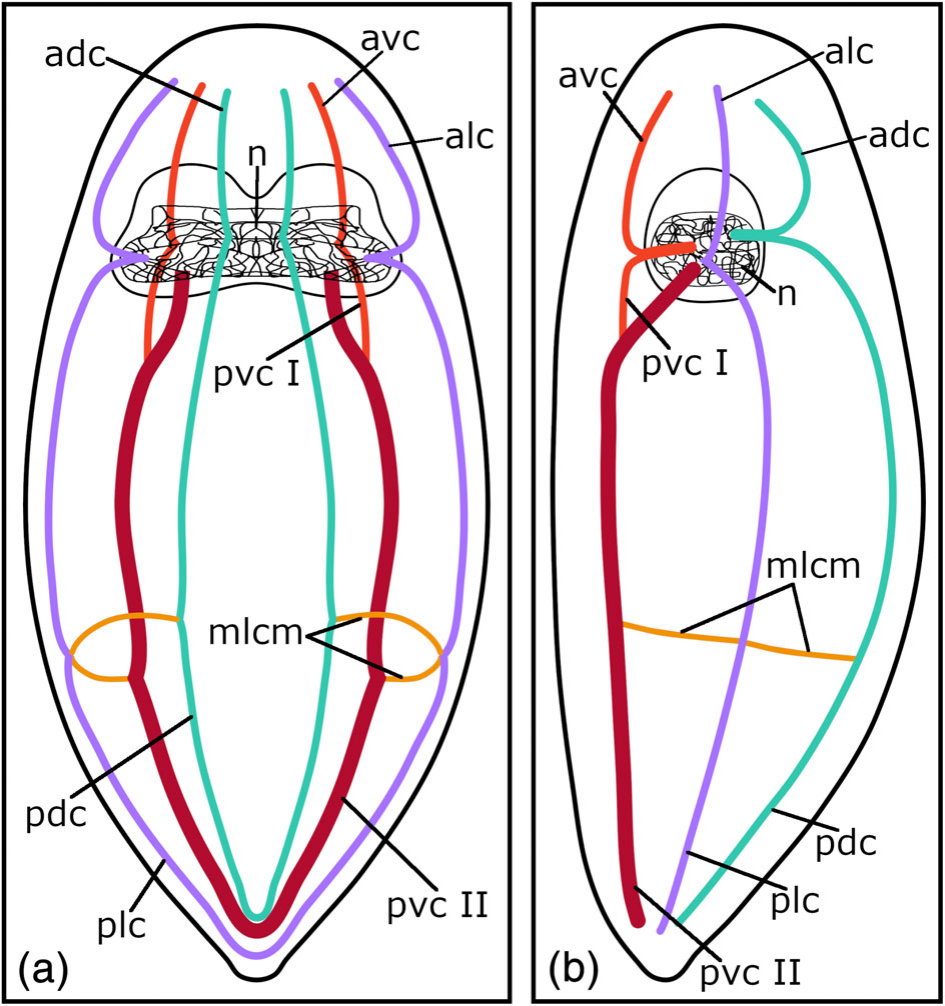
General pattern of the prolecithophoran 5‐HT‐IR orthogon in a (a) dorsal and a (b) lateral view. Anterior is up for both schemes, in (b) ventral is left. adc, anterior dorsal cords; alc, anterior lateral cords; avc, anterior ventral cords; mlcm, main lateral commissure; n, neuropil; pdc, posterior dorsal cords; plc, posterior lateral cords; pvc I, posterior ventral cords part I; pvc II, posterior ventral cords part II

The existence of a clade consisting of Prolecithophora, Fecampiida, and Tricladida has already been supported by several molecular studies (Laumer et al., 2015; Laumer & Giribet, 2014, 2017; Norén & Jondelius, 2002). However, no obvious synapomorphies, other than the fact that the species have opaque bodies, have been found that connect the three taxa (Norén & Jondelius, 2002). Also, the relationships within this clade are not resolved (Laumer & Giribet, 2017). While some studies position Tricladida as the sister group to Prolecithophora (Littlewood & Olsen, 2001; Norén & Jondelius, 1999), others support a clade of Fecampiida and Tricladida (Laumer & Giribet, 2014; Lockyer et al., 2003; Norén & Jondelius, 2002) and again another study suggests Prolecithophora as the sister group of Fecampiida (Laumer et al., 2015). Besides some specific differences, the present study shows a common pattern of the orthogon for prolecithophorans with the following characteristics: 1) three pairs of longitudinal nerve cords (dorsal, ventral, lateral) arise from the posterior part of the brain and split into an anterior and a posterior part, 2) the ventral nerve cords are the most prominent and they show a second root pair in the brain, 3) on either side of the posterior body part, the corresponding dorsal, ventral, and lateral nerve cords are connected to each other (Figure 7). A comparison of the orthogonal pattern with their close relatives has shown that fecampiids, in addition to the same three pairs of longitudinal nerve cords, also show a connection between the three longitudinal nerve cords of one body side. Triclads do not show close similarities with prolecithophorans, except that in both taxa the main cords show a ventral position. Based on the morphology of the serotonergic nervous system, our results suggest a closer affinity of Prolecithophora to Fecampiida. However, at this point it should be mentioned that some studies have shown that distantly related taxa have evolved similar types of orthogons independently and therefore it is supposed that the pattern of the orthogon is often related to the body shape and lifestyle of the animals (Joffe, 1990; Kotikova, 1991). Nonetheless, it can be noted that triclads always show the same pattern of the orthogon, regardless of their body size (Cebrià, 2007; Vila‐Farré et al., 2010).

## AUTHOR CONTRIBUTIONS

**Alexandra Grosbusch:** Conceptualization; investigation; methodology; validation; visualization; writing‐original draft; writing‐review and editing. **Philip Bertemes:** Conceptualization; investigation; methodology; visualization; writing‐review and editing. **Bernhard Egger:** Conceptualization; funding acquisition; investigation; methodology; project administration; supervision; validation; visualization; writing‐review and editing.

## CONFLICT OF INTERESTS

The authors declare that there is no conflict of interests.

### PEER REVIEW

The peer review history for this article is available at https://publons.com/publon/10.1002/jmor.21332.

## Data Availability

Data Availability Statement Data available on request from the authors.
